# Impaired fasting glucose levels among perinatally HIV-infected adolescents and youths in Dar es Salaam, Tanzania

**DOI:** 10.3389/fendo.2022.1045628

**Published:** 2022-12-06

**Authors:** Lilian Nkinda, Eliud Buberwa, Peter Memiah, Alieth Ntagalinda, Martin George, Frank Msafiri, Agricola Joachim, Mtebe Majigo, Kaushik Ramaiya, Bruno Sunguya

**Affiliations:** ^1^ Department of Microbiology an Immunology, School of Medicine, Muhimbili University of Health and Allied Sciences, Dar es Salaam, Tanzania; ^2^ Division of Epidemiology and Prevention: Institute of Human Virology, University of Maryland School of Medicine, Baltimore, MD, United States; ^3^ Department of Internal Medicine, Shree Hindu Mandal Hospital, Dar es Salaam, Tanzania; ^4^ Department of Community Health, School of Public Health and Social Science, Muhimbili University of Health and Allied Sciences, Dar es Salaam, Tanzania

**Keywords:** pre-diabetes, impaired fasting glucose, HIV, perinatal infection, adolescents, youth

## Abstract

**Objective:**

This study assessed impaired fasting glucose and associated factors among perinatally HIV-infected adolescents and youths in Dar es salaam Tanzania.

**Background:**

Impaired fasting glucose is a marker of heightened risk for developing type 2 diabetes among perinatally HIV-infected individuals. Therefore, identifying individuals at this stage is crucial to enable early intervention. Therefore, we assessed impaired fasting glucose (IFG) and associated factors among perinatally HIV-infected population in Dar es salaam Tanzania.

**Methods:**

A cross-sectional study was conducted among 152 adolescents and youth attending HIV clinic at Muhimbili National Hospital and Infectious Disease Centre from July to August 2020. Fasting blood glucose (>8 hours) was measured using *one-touch selects* LifeScan, CA, USA. We also examined C-Reactive Protein and interleukin-6 inflammatory biomarkers in relation to impaired fasting glucose (IFG). Associations between categorical variables were explored using Chi-square, and poison regression with robust variance was used to calculate the prevalence ratios.

**Results:**

Of the 152 participants, the majority were male (n=83[54.6%]), and the median age was 15(14-18) years. Overweight or obesity was prevalent in 16.4%, while more than one in ten (13.2%) had high blood pressure (≥149/90mmHg). All participants were on antiretroviral therapy (ART); 46% had used medication for over ten years, and about one in three had poor medication adherence. Among the recruited participants, 29% had impaired fasting glucose. The odds of IFG were two times higher in males compared to females (PR, 2.07, 95% CI 1.19 -3.59 p=0.001). Moreover, we found with every increase of Interleukin 6 biomarker there was a 1.01 probability increase of impaired fasting glucose (PR, 1.01, 95% CI 1.00 – 1.02 p=0.003).

**Conclusion:**

About one in three perinatally HIV-infected youths had impaired fasting glucose in Dar es Salaam, Tanzania, with males bearing the biggest brunt. Moreover, with every increase of 1.101 of the probability of having IFG increased. This calls for urgent measures to interrupt the progression to diabetes disease and prevent the dual burden of disease for this uniquely challenged population.

## Introduction

Impaired fasting glucose (IFG) is a type of pre-diabetes where an individual’s blood sugar levels during fasting are consistently above the normal range but below the diagnostic cut-off for type 2 diabetes mellitus (1). The burden of diabetes mellitus type 2 is higher in lower- and middle-income countries, and it is estimated that about a 90% increment of new cases will be from sub-Saharan Africa alone by the year 2030 (2). This increment will cripple economic progress and burden the country’s already overstretched health system. In addition, IFG has a higher predictive value for type 2 diabetes (3). Therefore, targeting those at the highest risk is the most cost-effective strategy for limited resources countries (4).

HIV-infected adults have a five-fold risk of developing diabetes mellitus compared to their HIV negative counter (5). It is also known that perinatally infected youth (acquired infection during pregnancy, birth or breastfeeding) have an excess risk of non-AIDS defining illnesses compared to adults or otherwise behaviorally HIV-infected youth and adolescents (6). Reports on the prevalence of insulin resistance, among HIV infected adolescents a condition preceding pre-diabetes and full-blown diabetes disease ranges from 6.5-34% (7–9). In contrast studies on perinatally infected adolescents and young adults evidences a higher rate (18-43% (10–12). The HIV infection on the immature immune system, long-term antiretroviral therapy (ART) exposure, and high HIV-related inflammation following poor adherence to ART are associated with this increased risk (13). As they transition to adulthood, the incidence of diabetes for this population is likely to surpass that of the current behaviorally infected adults.

Interventions and disease interrupters during the pre-diabetes stage can prevent or delay the occurrence of Type 2 diabetes (14). Disease interrupters such as rigorous screening, use of anti-inflammatory agents, and lifestyle changes have been implemented among the adult HIV population (15, 16). However, the burden of non-AIDS events is sparsely characterized among HIV-infected adolescents and youth, limiting the application of these interventions for this population. Furthermore, most diabetes risk behaviors, including poor diets and sedentary lifestyles, begin during adolescence (17). Therefore, reinforcing healthier choices and protective measures during this critical period can significantly change the health trajectory into adulthood and reduce the burden of comorbidity among the HIV population (17). However, evidence is unavailable on the burden and characteristics of IFG among perinatally HIV-infected youths in a high HIV prevalence context like Tanzania. This study was therefore conducted to address this gap.

## Methodology

### Study design and setting

This cross-sectional study was conducted among HIV-positive adolescents and young adults attending HIV care and treatment centers at Infectious Disease Center (IDC) and Muhimbili National Hospital (MNH), both located in Ilala district, Dar es salaam Tanzania. These facilities jointly serve 234 active adolescents and youth who attend from all the four districts of the region.

### Study population

The study population included HIV-infected individuals aged between 10 to 21 years who had fasted for more than 8 hours. Pregnant participants were excluded because of the possibility of pregnancy affecting glucose levels and participants on anti-inflammatory drugs in the last three months were excluded from this study, because it would interfere with the results inflammatory biomarkers levels.

### Sample size and sampling technique

The minimum sample size was 152 participants, calculated using Fisher’s formula. This study enrolled all adolescents who met the inclusion criteria and where willing to assented to participate in the study, but also had written informed consent from their parents. Informed consent for participants was obtained for participants 18 years old and above.

### Variables and measures

The outcome variable was Impaired fasting glucose, this was defined as fasting glucose levels above 6.1 but less than 7mmol/L (18). Body mass index (BMI) was weight divided by height square (kg/m²) and classified as underweight, overweight, and obese. Under-weight (BMI < 18 kg/m^2)^, normal (BMI ≥ 18 kg/m^2^ and ≤ 25 kg/m^2^), overweight (BMI > 25 kg/m^2^and < 30 kg/m^2^, and obese (BMI ≥ 30 kg/m^2^). In addition, blood pressure was considered high if BP ≥140/90mm/Hg (19). The adherence level at the last visit was extracted from the HIV ART card and recorded on a study tool as assessed by the attending Health care provider whether “GOOD”, MODERATE” or “POOR”. Good adherence referred to ≥95% adherence if less than three pills are missed, fair refers to 80–95% if 3–12 missed while poor adherence is less than 80% if 12 pills missed, all within one month of period (30days) (20). Smoking and alcohol consumption were determined if they had been used in the last 30 days from the day of interview. Occupation was asked to determine if the participants were still only students, or they were involved in income generating activities of moderate intensity (office work) or vigorous intensity causing excessive sweating (hard work). Physical activity was determined if one walked/cycled for at least 10 minutes daily (19).

### Data collection

The study used structured data collection tools (annex 1) to gather demographic characteristics such as age, sex, family history of diabetes (first degree relative), and behavioral risk factors like alcohol consumption and smoking. In addition, HIV-related factors included duration of ART use, ART adherence, and HIV viral load. Also, we conducted physical measurements for blood pressure, weight, and height. Blood pressure was measured twice on the left arm of the seated and relaxed participant using a sphygmomanometer. The body weight and height were measured in light clothes without footwear.

### Laboratory procedures

Four mls of EDTA blood was collected from every eligible adolescent. First, fasting glucose was measured using One-touch select ^©^ LifeScan, Inc AW 06716201A, whose 100% of glucose results of <100 mg/dL were within a bias of ±15 mg/dL, and 99.5% of results ≥100 mg/dL had a bias within the ±15% limit (21). Afterward, plasma was aliquoted and stored under −20°c awaiting inflammatory markers; Interleukin 6 and C-reactive Protein.

C-Reactive Protein and Interleukin 6 biomarkers were analyzed in the lab as described on this publication. CRP concentration above 3.1ng/liter will be considered high. The concentration of IL-6 beyond five pg/ml will be considered high (19).

### Statistical analysis

Descriptive statistics were performed, proportions were reported for categorical variables while median and interquartile range (IQR) for skewed data. To explore association for Impaired fasting glucose a chi-square test was used. The dependent variables considered for the analysis was in binary variables. For variables with the expected numbers of observations per cell less than 5, the fisher’s exact test was used in estimating the p-value. Variables with a p-value less than 0.2 were subjected to poison regression with robust variance to analyze the prevalence ratios. A p-value less than 0.05 was considered statistically significant.

## Results

### Participant characteristics

The characteristics of 152 study participants are summarized in **Table 1**. The median age was 15 years, with most of the participants being male (n=83[54.6%]) and 118 (77.6%) being students. Eighty-seven (57.2%) participants had completed their primary education. A total of 67(42.1%) had a family history of diabetes, 67(44.1%) were regularly performing physical activities, 7(4.6%) were smokers, and 16 (10.5%) were alcohol consumers. Twenty participants (13.2%) had high blood pressure, and 25(16.4%) were overweight/obese. About 45(29.6%) participants had poor ART adherence, 39(25.7%) had a high viral load above 5000 copies/ml, and 70(46.1%) participants had used antiretroviral drugs for above ten years.

**Table 1:** Characteristics of study participants .

Variable	Frequency, Median (IQR)	Percent (%)/mean (SD)
**Age**, median (IQR)	15 (14–18)	
**Age**		
10-14	60	39.5
15-19	62	40.8
20-24	30	19.7
**Sex**		
Male	83	54.6
Female	69	45.5
**Occupation**		
Student	118	77.6
Light work (moderate intensity)	22	14.5
Hard work (Vigorous Intensity)	12	7.9
**Education status**		
Ongoing primary education	5	3.3
Finish primary education	87	57.2
Secondary school and above	60	39.5
**Family history of diabetes (first degree)**		
Yes	67	42.1
No	88	57.9
**Physical activity (**walk/cycle,>10mins daily)		
Yes	67	44.1
No	85	55.9
**Smoking status (**last 30 days**)**		
Yes	7	4.6
No	145	95.4
**Alcohol consumption (**last 30 days**)**		
Yes	16	10.5
No	136	89.5
**ART adherence**		
Good (>95%)	74	48.6
Moderate (80–95)	33	21.7
Poor (<80%)	45	29.6
**Viral load (copies/ml)**		
1000 – 5000	113	74.3
Above 5000	39	25.7
**Viral load, median (IQR)**	2972.5 (2126.5 - 5194.3)	
**ART duration (years)**		
>10	70	46.1
6 -10	48	31.6
0 -5	34	22.3
ART duration, median (IQR)	9 (6–13)	
**BMI**		
Underweight + normal	127	83.6
Overweight + obese	25	16.4
**BMI**, median (IQR)	20.9 (18.7 - 23.5)	
**High blood pressure**		
Yes	20	13.2
No	132	86.8
SBP (mmHg), median (IQR)	118 (117–126)	
DBP (mmHg), median (IQR)	78 (76-81.25)	

BMI, Body Mass Index; SBP, Systolic Blood Pressure; DBP, Diastolic Blood Pressure.

### Impaired fasting glucose, interleukin 6, and C-reactive protein

The proportion of participants with impaired fasting glucose was 28.9% (n=45). High IL6 and High CRP were observed in 34 (22.4%) and 78 (51.30%) participants, respectively. Thirteen (8.6%) participants had impaired fasting glucose and high IL6 levels. Likewise, impaired fasting glucose and high levels of CRP were observed in 26(17.1%) (**Table 2**).

**Table 2:** Proportion of impaired fasting glucose, high IL6, and high CRP (n = 152).

Variable	Frequency	Percent (%)
Impaired fasting glucose (IFG)	45	29.6
High IL6	34	22.4
IFG with high IL6	13	8.6
High CRP	78	51.3
IFG with high CRP	26	17.1
IL6 – Interleukin 6, CRP – C-reactive protein		

### Factors associated with impaired fasting glucose

Among the analyzed factors, only gender was significantly associated with IFG (p=0.008). Therefore, all variables with a p-value ≤0.2, which were family history of diabetes, physical activity, alcohol consumption and IL6 inflammatory biomarker, were subjected to poison regression analysis with robust variance. BMI and viral load had high multicollinearity (R=0.88) hence only one of the variables (BMI) was subjected to the model with other factors. The odds of having IFG were two times higher in males compared to female (PR, 2.07, 95%CI, 1.19 – 3.59 p=0.001) Moreover, with every increase of 1.101 of IL6 the probability of having IFG increased (PR, 1.01, 95 CI (1.00 – 1.02 p=0.003) (**Table 3**).

**Table 3:** Bivariate and Multivariate analysis of factors associated with impaired fasting glucose.

**Variable**	**Impaired fasting glucose** **N (%)**	**Normoglycemia** **N (%)**	**p-value**	**PR (95% CI)**	**p-value**
**Sex**					
Female	13 (18.8)	56 (81.2)	0.008	1	
Male	32 (38.6)	51 (61.4)		2.07 (1.19 – 3.59)	0.010
Age					
10-14	17 (28.3)	43 (71.7)			
15-19	16 (25.8)	46 (74.2)			
20-24	11 (36.7)	19 (63.3)	0.581		
**Physical activity**					
Yes	23 (34.3)	44 (65.7)	0.201	1	
No	22 (25.9)	63 (74.1)		0.81 (0.50 – 1.30)	0.352
**Smoking status**					
Yes	3 (42.9)	4 (57.1)	0.421	–	
No	42 (29.0)	103 (71.0)			
**Alcohol consumption**					
No	43 (31.6)	93 (68.4)	0.152	1	
Yes	2 (12.5)	14 (87.5)		0.44 (0.18 – 1.66)	0.232
**Family History of diabetes type 2**					
No	23 (26.1)	65 (65.6)	0.211	1	
Yes	22 (34.4)	42 (43.4)		1.21 (0.79 – 1.97)	0.377
**SBP (mmHg)(IQR)**	118 (117, 130)	118 (117, 123)	0.558	–	
**DBP (mmHg)(IQR)**	79 (76, 87)	78 (76, 80)	0.438	–	
**High blood pressure**					
Normal	30 (27.3)	77 (70.0)	0.972	–	
Pre-hypertension	9 (40.9)	13 (59.1)			
Hypertension	6 (30.0)	14 (70.0)			
**ART duration**					
0 -5	12 (35.3)	21 (61.8)	0.583	–	
6 -10	15 (31.3)	31 (64.6)			
>10	18 (25.7)	52 (74.3)			
**ART adherence**					
Good	22 (29.7)	52 (70.3)	0.682	–	
Moderate	8 (24.2)	25 (75.8)			
Poor	15 (33.3)	30 (66.7)			
**Occupation**					
Student	35 (29.7)	80 (67.8)	0.931	–	
Light work	6 (27.3)	16 (72.7)			
Hard work	4 (33.3)	8 (66.7)			
**BMI (IQR)**	20.3 (18.5,22.6)	21.3 (18.7,23.6)	0.147	0.97 (0.90-1.05)	0.48
**BMI**					
Underweight	10 (38.5)	15 (57.7)	0.840		
Normal	28 (27.7)	71 (70.3)		–	
Overweight	7 (29.7)	17 (70.8)			
Obese	0 (0.0)	1 (100.0)			
**Viral load (IQR)**	2670 (2004,3830)	3166 (2304,5775)	0.132	–	
**Viral load**					
	44 (29.1)	104 (68.9)	0.301		
	1 (100.0)	0 (0.0)			
**IL6 level**	32 (21.1)	85 (55.9)	1.011		
	12 (7.9)	23 (15.1)			
**IL6 level (IQR)**	3.6 (1.94,5.10)	3.38 (0.13,4.80)	0.154	1.01 (1.00 – 1.02)	0.003
**CRP level**					
Normal	19 (25.7)	53 (34.9)	0.302	–	
High	26 (33.3)	55 (36.2)			

## Discussion

The current study revealed a relatively high burden of impaired blood glucose among perinatally HIV-infected youth in Dar es Salaam, Tanzania. In addition, our study observed that around 13% of participants have high blood pressure (≥149/90mmHg), and nearly one-third have poor adherence to antiretroviral therapy. Both conditions are important risk factors for the early onset of non-communicable diseases, a growing burden in the country. Moreover, with every increase of 1.101 of IL6 the probability of having IFG increased and the males were twice more likely to have IFG compared to females.

HIV perinatally infected youth are currently living for many years since the introduction of antiretroviral therapy (18). However, several studies have observed glucose impairment ranging from insulin resistance (8–10) to pre-diabetes (11, 12), and even full-blown diabetes disease (9). The current study observed that close to one in three youths (29%) had impaired fasting glucose, but the affected youths were unaware of their status, which further increase the risk of poor disease progression. Data from sub–Saharan Africa(SSA) have shown IFG to range from 2-6% (8, 22, 23). These results are comparatively lower than the current study. Although the difference could be attributed by the lower mean age of 9-12 years or mean duration of ARV use of 4-6 years compared to the current study where the mean age is 16 years and 10years respectively. Data from a Hispanic population with similar mean age (15 years) and ART duration (11years) reported IFG of 4.6% (24). Given that the occurrence of glucose alterations is multifactorial, these varying results across different geographical regions could reflect the varying dietary habits, genetic attributes and ethnicity (24, 25). Moreover, from the Findings from Latin America and Europe have shown the prevalence of IFG to range from 0.4-7% (26–29), while reports from Asian communities range from 2.6-43% (9, 11, 12). This necessitates for population specific data, to make informed decisions.

It is expected to observe a higher burden of diabetes and pre-diabetes in adults than in adolescents and youth (23); however, the prevalence in the current study is comparable to the prevalence of Pre-diabetes reported among adult PLWH in sub-Saharan Africa, which ranges from 15-27% (3, 5, 19). Despite the growing concerns about the increasing burden of impaired glucose metabolism among the HIV population (5, 19), no specific preventive measure has been incorporated into HIV care and treatment in Tanzania. The perinatally HIV-infected individuals will have a lifetime exposure to ART and HIV-related inflammation, hence have a greater risk of succumbing to the dual burden of disease at a younger age (16, 30) if no measures are taken. To the best of our knowledge this is among the pioneer study reporting IFG among perinatal infected adolescent and youth in Tanzania.

Moreover, we found with every increase of Interleukin 6 biomarker there is a 1.01 probability increase of IFG. Even with effective ART, chronic inflammation persists and has been linked the incidence of insulin insensitivity and eventually diabetes mellitus type 2 (3, 19). Several findings have shown incidence of diabetes following high levels of IL6 among other markers such as CRP (31, 32). Whether IL6 can predict the incidence of diabetes for the HIV population is still not clear, as the findings are highly heterogenous (19, 31). According to Farmer et al. the predictability of a marker is also influenced by the genetic variability and ethnicity, therefore more studies on a similar population are warranted to establish a reliable marker for the adolescent and youth population (33). The current study also measured CRP levels among the participants, but the findings were not significantly correlated.

Being male increased the risk of IFG by two-fold in this study. Similar findings have been reported elsewhere for the general population and PLHIV infection (5, 34). Contrasting findings argue that glucose impairment fluctuates depending on the life stage; where females are reported to have higher rates of glucose disturbances during youth, while males have higher odds at mid-life (35). A comparably smaller sample size could in-part explain the variation of these findings since a large study (n=4.4m) involving 146 countries reported men to have a higher prevalence of glucose-impaired metabolism independent of age (36). The other explanation could be the modality of glucose screening, where findings from a large European Diabetes Epidemiology study confirmed male predominance when fasting blood is used for diagnosis but not when a 2hour plasma glucose is tested after oral glucose load (37). Therefore, these findings could be an underestimation of the actual burden.

This study reports hypertension being prevalent among adolescents and youth (13%) who were perinatally infected with HIV. This is lower than another study conducted among adult perinatally infected subjects (30%) with a median age of 26 years (38). The age gap could have influenced this difference, as evidenced in the Baltimore study that reported a prevalence of hypertension of 26% among young adults, and the incidence significantly increased as the population reached adulthood (<18 years) (39). Factors at play for the perinatally HIV-infected individuals are not characterized, most probably because of the multifactorial pathophysiology (40). However, structural changes in the cardiac vasculature are observed as early as the first ten years of life for vertically HIV-infected subjects (41). Poorly controlled hypertension may eventually lead to cardiovascular diseases, kidney diseases, and mortality (42); hence early identification of high-risk individuals through screening is critical to prevent the development of these outcomes.

Having Poor ART drug adherence was not significantly associated with having IFG, although 29% were reported to have poor adherence to medication. This is regarded as a health risk behavior as it endangers the life and livelihood of the concerned and increases the risk of HIV transmission (43). Similar to the current study, sub-optimal ART adherence is reported in several studies among perinatally HIV-infected individuals (44, 45), where several factors have been correlated to contribute, such as the child’s age, developmental stage, and psychosocial state, among others (46). The dual burden of HIV and non-AIDS chronic disease could further complicate HIV care and treatment outcomes, including adherence to care; hence, youth-friendly adherence counseling is necessary to reduce adherence barriers for this uniquely challenged population (12, 46).

About 16% of our study participants were overweight or obese, but this was not significantly associated with impaired fasting glucose in our study. Although, it is a well-documented risk factor influencing glucose metabolism impairment and cardiovascular disease among the HIV population (3, 34). Similar findings have been reported in other studies, where a significant correlation was not attained (5, 19). The lower proportion of this variable may have limited the our ability to detect such associations.

Of note, we observed a trend of a non-significant low proportion of IFG in those with physical activity. According to Kemps et al, physical training can improve insulin sensitivity and overall sugar levels (47). Not being physically active could be influenced by being an urban resident, this is also reported in several studies that compared urban and HIV rural populations (48). Interestingly being physically active is reported to improve ART adherence (49). This could be because of its potential to improve one’s mood and alienate depressive symptoms (49), although further studies are required on this phenomenon to justify incorporating physical exercise in HIV care and treatment for perinatally infected individuals.

This study is not without limitation: this includes the use of a point of care device to measure glucose levels as this may overestimate the burden glucose impairment (21). None the less these are the devices used for screening and diagnosis of diabetes in SSA (50). Furthermore, the puberty stage has been associated with insulin insensitivity, however we did not study the role of puberty in relation to impaired fasting glucose because of the challenges in assessing this variable using tanner staging scale in the midst of provision of HIV care and treatment routine services.

## Conclusion

The prevalence of IFG was high among HIV perinatally infected youths, found more in males than females. Furthermore increase of 1.101 of IL6 increased the probability of having IFG. Therefore, this study recommends incorporating scheduled glucose screening to identify these individuals early before developing full-blown diabetes. Furthermore, this study calls for urgent measures to interrupt the progression to diabetes to prevent the dual burden of disease for this uniquely challenged population.

## Data availability statement

The original contributions presented in the study are included in the article/**Supplementary Material**. Further inquiries can be directed to the corresponding author.

## Ethics statement

The studies involving human participants were reviewed and approved by Senate of Research and Publication Committee and the Institutional Review Board of Muhimbili University of Health and Allied Sciences (MUHAS). Written informed consent to participate in this study was provided by the participants’ legal guardian/next of kin. Written informed consent was obtained from a parent/legal guardian as well as the Nurse Matron for those below the age of 18. In addition, permission to be involved in the study was obtained from the child himself/herself. For children above the age of 18 years written informed consent was obtained from all subjects before the study began.

## Author contributions

LN conceptualized the study. Methodology and data collection were done by EB, AN, MG. FM and MM performed data analysis. LN developed the initial manuscript draft. AJ, PM, BS and KR critically reviewed the manuscript. All authors have read and approved this manuscript. This manuscript has been submitted solely to this journal and is not published or submitted elsewhere.
