# Supplementary material for: Impaired fasting glucose levels among perinatally HIV-infected adolescents and youths in Dar es Salaam, Tanzania
Source: Front Endocrinol (Lausanne). 2022 Dec 6;13:1045628. doi: 10.3389/fendo.2022.1045628 (PMC9763284; doi:10.3389/fendo.2022.1045628)
Supplement: Supplementary file 1 [file Table_1.docx]

## **Questionnaire (English Version)**

**Impaired fasting glucose among HIV perinatal infected adolescents and youth in Dar es Salaam Tanzania.**

Patient ID……………………... Date …………………….

Phone number…………………….

Time of Last meal: ………….

**SOCIAL INFORMATION**

1. Gender …………
2. Age (dd/mm/yy)…………

| 3. | | | Family history of diabetes | | | | | | |
| --- | --- | --- | --- | --- | --- | --- | --- | --- | --- |
| First degree (father/mother) | | | | | | Secondary relatives: | | | |
| 4. | | | Highest level of education: | | | | | | |
| Has not completed primary: | | | | Completed primary: | | Secondary and above: | | | |
| 5. | | Marital status | | | | | | | |
| Single: | | | | Married: | | Other: | | | |
| 6; | | | Type of work | | | | | | |
| Hard labor, Cause excessive sweating: | | | | Office/Light work: | | student: | | | |
| **BEHAVIOURAL RISK FACTORS** | | | | | | | | | |
| 7. | Physical activity | | | | | | | | |
| Walking/cycling > 10mins daily: Yes No | | | | | | | | | |
| 8. | | Smoking | | | | | | | |
| Current smoker (30 days)  Cigarettes per week…………………… | | | | | Ever smoked (>12 months) | | | | Never smoked |
| 9. | | Fruits and vegetables for the week | | | | | | | |
| None: | | | | | Once a week | | More than once a week: | | |
| 10. | Alcohol consumption | | | | | | | | |
|  | Current drinker (30 days)  Bottles per week: ………………. | | | | Ever drank (>12 months) | | | Never Drank | |

**INFORMATION ON BODY MEASUREMENTS**

| 12. | | | | Blood pressure measurement: | | | | | | | | | |  |
| --- | --- | --- | --- | --- | --- | --- | --- | --- | --- | --- | --- | --- | --- | --- |
|  | | | | Weight in kilograms: ________ | | | | Height in meters: _______ | | | | | |  |
| 13 | | | | BMI (kg/$m^{2}$) | | | |  | | | | | |  |
| ≤30kg/$m^{2}$: | | | | | (<25 to<30) kg/$m^{2}$: | | |  | | | (18 -25) kg/$m^{2}$: | | <18 kg/$m^{2}$: | |
| 14. | | | Waist thickness: ____________ | | | | | | | | | | |  |
| M: >84cm: | | | | | | <84cm: | | | | K: >94cm: | | <94cm: | |  |
| **INFORMATION FROM FILE** | | | | | | | | | | | | | |  |
| 15 | CD4 before starting ART: ___________________ | | | | | | | | | | | | |  |
| <200cells/µl: | | | | | | | <200cells/µl: | | | | | >350 cells/µl: | |  |
| 16. | | Recent CD4 count: ………………… | | | | | | | | | | | |  |
| 17. | | The type of ART being uses……………………. | | | | | | | | | | | |  |
| 18. | | The time spent on ART: …………………… | | | | | | | | | | | |  |
| 19 | | Recent viral load: ………………………. | | | | | | | | | | | |  |
| 20 | | ART Adherence | | | | | | | | | | | |  |
| Good (missed <3pills) : | | | | | | | | | Moderate (missed 3-12 pills: | | | Poor (missed >12 pills): | |  |

21. Recent Opportunistic diseases ………………………………………………..

22. Have you taken any other medicine other than ARV to prevent infection………………………….. To prevent inflammation……………………any other……………….

***Report Glucose levels on the lab form.**
